# Identification of novel and known variants in *GAA, MYH6, NEXN, ATA3A, RHBDF1,* and *ACTA1* genes in coronary artery disease patients by Whole-exome sequencing

**DOI:** 10.3389/fcvm.2026.1853197

**Published:** 2026-09-04

**Authors:** Rashid Mir, Mohammad Fahad Ullah, Jamsheed Javid, Imadeldin Elfaki, Faisal H. Altemani, Jameel Barnawi, Naseh A. Algehainy, Mohammed M. Jalal, Malik A. Altayar, Aadil Yousif, Eram Hussain, Faris J. Tayeb, Faisel M. AbuDuhier, Salem Owaid Albalawi, Syed Khalid Mustafa

**Affiliations:** 1 Prince Fahad Bin Sultan Chair for Biomedical Research, University of Tabuk, Tabuk, Saudi Arabia; 2 Department of Medical Laboratory Technology, Faculty of Applied Medical Sciences, University of Tabuk, Tabuk, Saudi Arabia; 3 Department of Biochemistry, Faculty of Science, University of Tabuk, Tabuk, Saudi Arabia; 4 Department of Cardiology, King Fahd Specialist Hospital, Tabuk, Saudi Arabia; 5 Department of Chemistry, Faculty of Science, University of Tabuk, Tabuk, Saudi Arabia

**Keywords:** cardiovascular health, coronary artery disease, genetic variations, human phenotype, whole exome sequencing

## Abstract

**Background:**

The etiology of CAD is multifactorial, involving a complex interplay of genetic, environmental, and lifestyle factors, and CAD markedly increases the likelihood of impaired quality of life.

**Aim:**

To identify potentially impactful genetic variants in patients with CAD that may influence the risk, severity, and clinical outcomes of the condition, we performed whole exome sequencing (WES) in 28 patients. Diverse risk factors such as gender and clinical conditions, including hypertension, hyperlipidemia, obesity, and T2DM, which are known contributors to cardiovascular adversities, have been found to be present in a significant proportion of the patients.

**Results:**

Our study identified 13,289 variants in 108 genes across the cohort, with a median of 545 per patient. The most frequently mutated genes were TTN, followed by GAA, SVIL, NRAP, and DMD, all of which are related to sarcomere structural and functional integrity and cardiomyopathies. Analysis of VEP impact identified 341 variants in 58 genes with 122 novel variants (36%) not previously reported in public databases. The variants were classified as missense (66%), followed by frameshifts (25%) and in-frame indels (5%), with a minor proportion (4%) of nonsense, splice-site, and other protein-altering variants. Among these 58 genes, TTN emerged as the most frequently mutated gene, present in 96% of patient samples, while the other substantially mutated genes in patients included FLNC (29%), AGL (25%), ALPK3 (21%), and DSP (21%). A comprehensive analysis of candidate variants identified 21 variants classified as either pathogenic (3) or likely pathogenic (18), which may play a significant role in the development of cardiovascular disease. These variants met the American College of Medical Genetics and Genomics (ACMG) criteria for PM2 classification, indicating a very low (<1%) or absent frequency in population databases. Among these, eight variants were novel and classified as likely pathogenic, reported in genes GAA, MYH6, NEXN, ATA3A, RHBDF1, and ACTA1. Most gene variants have functional significance in cardiovascular pathologies, as demonstrated by gene ontology and human phenotype ontology enrichment analyses reported in this study.

**Conclusion:**

The study has utilized whole exome sequencing to identify both known and novel gene variants that may contribute to disease risk, facilitating a deeper understanding of the genetic landscape of CAD. This high-resolution approach allows the detection of pathogenic and likely pathogenic variants in critical genes associated with cardiovascular health and provides evidence of functional significance, thereby elucidating complex interactions between genetic factors and enhancing understanding of disease mechanisms.

## Introduction

Coronary artery disease (CAD) represents a significant challenge to global health, with its prevalence rising alarmingly across diverse populations. Cardiovascular diseases, which include CAD, stand as a foremost contributor to morbidity and mortality across the globe, ranking as one of the leading causes of death in both developed and developing nations. According to the World Health Organization (WHO), in 2019 cardiovascular diseases, which include CAD, were responsible for approximately 32% of global deaths, translating to around 17.9 million individuals annually. The data also reported that out of the 17 million premature deaths (under the age of 70) due to non-communicable diseases in 2019, 38% were caused by CVDs (WHO factsheet) (1). In 2021, CVDs accounted for 20.5 million deaths, comprising approximately one-third of all global deaths (2). CAD has potential complications and poses a significant risk of events ranging from mild discomfort to fatal outcomes, including chest pain, myocardial infarction, arrhythmias, heart failure, and sudden cardiac death (3). Coronary artery disease is often accompanied by a range of comorbid conditions that can significantly exacerbate the symptoms and severity of CAD, leading to poor prognosis. The etiology of CAD is multifactorial, involving a complex interplay of genetic, environmental, and lifestyle factors. Traditional risk factors, including hypertension, hyperlipidemia, diabetes mellitus, tobacco use, obesity, and physical inactivity, have been associated with an increased likelihood of CAD, markedly impairing the quality of life. These factors contribute to the pathophysiological processes that lead to atherosclerosis, the underlying condition of CAD, by damaging the endothelial lining of blood vessels and promoting inflammation. Individuals suffering from Type 2 diabetes mellitus (T2DM) face an increased likelihood (2–4-fold) of developing coronary artery disease compared to those who do not have this condition (4). This heightened risk is primarily due to the metabolic and vascular complications associated with diabetes, which can lead to the early onset of atherosclerosis and other cardiovascular issues. It has been recorded that approximately 80% of patients with T2DM ultimately succumb to complications related to cardiovascular diseases, with CAD being a significant contributor to these fatalities (5). Studies indicate that patients with T2DM tend to have coronary plaques characterized by larger necrotic cores and heightened inflammation, featuring increased numbers of T lymphocytes and macrophages. Additionally, these patients exhibit a higher rate of positive remodeling and plaque ruptures compared to non-diabetic individuals, indicating a more aggressive atherosclerotic process (6). Studies using advanced genome-wide techniques have demonstrated that a substantial portion of the genetic component of CAD is attributable to common alleles at many loci, estimating CAD heritability at 35%–50% (7). Additionally, the Framingham Heart Study found that having a family history of cardiovascular disease in a parent or sibling was a significant predictor of developing the disease (8, 9). It has been observed in genome-wide association studies (GWAS) that the majority of common inter-individual genetic variations can be analyzed individually by comparing their frequency in disease cases and healthy controls (10). However, certain rare variants that are not usually identified in GWASs have also been reported in certain genes such as *SCARB*, *LDLR*, *APOA*, *DYRK1B*, *MEF2A*, and *PDE5A*, which cause a monogenic form of CAD (11, 12). It is understood that genetic information offers the only tool to guide primordial prevention (i.e., targeted interventions) before onset of traditional risk factors or visible disease manifestations. This can effectively identify subjects at risk with greater precision than conventional risk scores by using a polygenic risk score that analyzes and predicts the outcome at birth (13). Traditional genetic investigations of CAD primarily focus on canonical pathways involved in lipid metabolism, plaque stability, and systemic inflammation. Nevertheless, a substantial subset of CAD patients experiences acute ischemic events, severe myocardial remodeling, or sudden cardiac death, in which secondary structural or electrophysiological vulnerabilities significantly increase baseline risk. To investigate this genetic overlap, the present study employs whole-exome sequencing in conjunction with a comprehensive multi-disease super-panel approach. By examining genetic variations across integrated structural and electrical cardiac sub-panels, the study seeks to identify rare, high-impact modifiers that influence myocardial resilience during severe ischemic stress, thereby extending beyond conventional atherosclerotic risk metrics. Through this combined approach, we identified novel genetic variants that had not been previously documented.

## Methodology

### Subjects

The present study was conducted in accordance with the guidelines outlined in the Helsinki Declaration and was ethically approved by the University of Tabuk Research Ethics Committee (# UT-91-23-2020). A total of 28 clinically confirmed CAD patients were included in this study. All the participants signed the consent form. We included patients from the King Fahad Specialist Hospital, Tabuk, KSA, who underwent elective angiography for diagnosis of stable angina. The following tests were conducted: x-rays, exercise stress testing, myocardial perfusion imaging, ambulatory electrocardiography, Holter monitoring, chest echocardiogram, computed tomography coronary angiography, and multigated acquisition scans (MUGA). CAD was defined based on clinical symptoms (stable or unstable angina) and confirmed through invasive coronary angiography. Inclusion criteria specified macrovascular disease with at least 50% luminal diameter stenosis in one or more of the three major coronary arteries: left anterior descending (LAD), left circumflex (LCx), or right coronary artery (RCA). A total of 28 patients were consecutively selected from a larger registry to provide a representative sample of symptomatic obstructive CAD. Although electrocardiograms (ECG) and echocardiograms were performed as part of the initial diagnostic process for stable angina, the only inclusion criterion was structural CAD, defined as stenosis of 50% or greater. Patients were not pre-screened to exclude those with primary rhythm or myocardial disorders.

### Genomic DNA preparation and sequencing

High-quality human genomic DNA was used as the starting material for this study. Library preparation was performed following the protocol outlined in the Twist Human Core Exome 2.0 Kit instruction manual. Sequencing was conducted on the Illumina NovaSeq 6000 platform according to the manufacturer's guidelines.

### Quality control and preprocessing of sequencing data

The quality of the raw sequencing reads was assessed using FastQC (v0.11.9) (14) to ensure reliable data. Reads were then processed to remove sequencing adapters and low-quality bases using TrimGalore (v0.6.6), providing high-quality (HQ) reads for downstream analysis. Using a standard mapping protocol, the resulting HQ reads were aligned to the human reference genome (hg38).

### Variant calling and annotation

The variant discovery process was conducted using the Genome Analysis Toolkit (GATK) v4.3 (15), following the best practice pipeline. HaplotypeCaller was employed to identify small variants, including single nucleotide variants (SNVs) and short insertions/deletions (InDels). Quality Score Recalibration (VQSR), with tranche sensitivity set to 99.5% for SNPs and 99.0% for indels. Hard filtering was applied to variants failing VQSR using stringent thresholds (QD < 2.0, FS > 60.0, and MQ < 40.0 for SNPs). Our final callset exhibited a Transition-to-Transversion (Ti/Tv) ratio of 1.99, an average variant call rate of 99%, and a mean on-target depth of ∼91X, with >95% of target bases covered at >20×.

Identified variants were annotated comprehensively using various databases and tools to gain biological and clinical insights. For gene identification and variant characterization, the RefSeq database was utilized. The Ensembl Variant Effect Predictor (VEP) was used to annotate variants, providing detailed information on their functional and biological consequences (16). Default VEP annotations were supplemented with several plugins, including dbNSFP (17), CADD (18), and Phenotypes (19), to enhance the annotation quality. Potential disease associations of variants were explored through publicly available databases such as OMIM (20), ClinVar (21), and UniProtVar (22). Population allele frequency data were sourced from the 1,000 Genomes Project (23) and gnomAD (24) (including both exome and genome datasets) to distinguish rare variants from common polymorphisms effectively. For functional prediction of mutations, tools integrated within dbNSFP such as SIFT (25), PolyPhen (26), FATHMM (27), MutationTaster (28), MutationAssessor (29), and PROVEAN (30) were employed. Missense Variants were further annotated using CADD scores. The SIFT-indel tool (31) was applied to evaluate the functional impact of InDels. Additionally, only canonical transcript-dependent consequences were retained in the final VEP-annotated file to ensure consistency and relevance in downstream analyses.

### Filtering and classification of variants

To systematically identify rare variants influencing cardiac structure and electrical stability in obstructive coronary artery disease (CAD), we employed the comprehensive “Sudden Unexplained Death/Cardiac Event” panel from the Genomics England PanelApp (32). This framework functions not as a single disease entity but as a high-density super-panel, enabling evaluation of genetic variation across eight distinct, clinically curated cardiovascular sub-panels:

1. Hypertrophic cardiomyopathy
2. Catecholaminergic polymorphic ventricular tachycardia
3. Dilated and arrhythmogenic cardiomyopathy
4. Short QT syndrome
5. Arrhythmogenic right ventricular cardiomyopathy
6. Brugada syndrome and cardiac sodium channel disease
7. Long QT syndrome
8. Progressive cardiac conduction disease

By screening our whole-exome sequencing data using this unified architecture, we systematically evaluated the presence of subclinical sarcomeric, desmosomal, and ion-channel variants that may influence phenotypic severity or reduce the threshold for severe ischemic complications.

Variants within these genes were prioritized for further analysis. To ensure the clinical relevance of the identified variants, we applied a multi-step filtering process. Variants with a population frequency of 1% or less in databases like gnomAD and 1,000 Genomes Project were retained. The analysis was restricted to protein-coding regions and canonical splice sites, prioritizing variants with “HIGH” or “MODERATE” VEP impact. To assess the potential clinical implications of the filtered variants, we employed a comprehensive annotation approach. Variants were annotated using clinical databases like ClinVar and UniprotVar to leverage existing clinical knowledge. Additionally, a combination of standalone and ensemble in silico prediction tools, including SIFT, PolyPhen-2, FATHMM, MutationTaster, Mutation Assessor, CADD, PROVEAN, and SIFT-Indels, were used to predict the impact of nonsynonymous variants, indels, and frameshift variants. Based on the combined information from clinical databases and in silico predictions, variants were classified into five categories: Benign, Likely Benign, Variant of Unknown Significance, Likely Pathogenic, and Pathogenic. The classification process prioritized clinical data over in silico predictions. In cases of conflicting clinical annotations, in silico predictions were used to make a final classification. For variants absent in clinical databases, stringent criteria based on CADD scores (≥20 for deleterious) and in silico tool predictions (at least 4 tools having consensus) were applied to classify them as Likely Pathogenic or Likely Benign. Variants that did not meet these criteria were classified as VUS.

Novelty of each variant was assessed by cross-referencing identified alterations with major public genomic repositories, such as dbSNP, ClinVar, gnomAD, COSMIC, and the 1,000 Genomes Project. For robust classification, an exhaustive PubMed literature search was conducted for each of the eight novel, likely pathogenic variants. None of these specific genomic coordinates have been previously reported in peer-reviewed case reports or cohort studies.

### Functional annotation of candidate variant genes

To elucidate the functional impact of the identified pathogenic and likely pathogenic variants, we employed the g:profiler webserver (33) for enrichment analysis. This tool enables the systematic exploration of gene sets, identifying overrepresented Gene Ontology (GO) terms. By focusing on Gene Ontology—Molecular Function (GO:MF) and Biological Process (GO:BP) terms, as well as Human Phenotype ontology, we aimed to uncover the specific molecular and cellular processes influenced by these variants and their potential contribution to cardiovascular disease. Multiple testing corrections were controlled using the default g:SCS (Set Counts and Sizes) threshold algorithm, and only terms with an adjusted *p*-value (p_adj_ < 0.05) were considered statistically significant. In contrast to standard independent correction methods such as Bonferroni or Benjamini-Hochberg, g:SCS calculates a more stringent, analytically derived significance threshold (*α* = 0.05). This approach accounts for the complex, hierarchically dependent network structure characteristic of Gene Ontology (GO) and Human Phenotype Ontology (HPO) terms, thereby minimizing type I error while preserving biological signal.

## Results

### Study population characteristics

A total of 28 patients diagnosed with coronary artery disease were enrolled in this study. The median age of the participants was 53 years [interquartile range (IQR): 40–63], with a male predominance (86%). A significant proportion of patients had hypertension (54%) and hyperlipidemia (46%). Additionally, 54% were overweight or obese, and 54% were diabetic. In terms of angina presentation, 71% had stable angina, while 29% had unstable angina. A history of myocardial infarction (MI) was reported in 36% of the participants, with 14% experiencing ST-elevation myocardial infarction (STEMI), 14% experiencing non-ST-elevation myocardial infarction (NSTEMI), and 3.6% undergoing coronary artery bypass grafting (CABG). A familial history of cardiovascular disease was present in 36% of the participants. Laboratory parameters revealed a median cholesterol level of 4 mmol/L (IQR: 3–127), a median triglyceride level of 3 mmol/L (IQR: 1–160), a median LDL-C level of 90 mmol/L (IQR: 80–121), and a median HDL-C level of 1 mmol/L (IQR: 1–24). The median creatinine level was 71 μmol/L (IQR: 2–118), and the median hemoglobin level was 14.75 g/dL (IQR: 12.57–16.33). Table 1 enlist the patient characteristic of the study cohort.

**Table 1:** Clinical characteristics of the study cohort.

Characteristic	Coronary artery disease (CAD) Patients (*N* = 28)
	Median (IQR); *n* (%)
Age (Years)	53 (40–63)
Gender	
Female	4 (14%)
Male	24 (86%)
Hypertension	15 (54%)
Hyperlipidemia	13 (46%)
Overweight/Obesity	
Unknown	1 (3·6%)
No	12 (43%)
Yes	15 (54%)
Diabetes Mellitus	15 (54%)
Angina	
Stable	20 (71%)
Unstable	8 (29%)
Myocardial Infarction (MI)	
No	18 (64%)
CABG	1 (3·6%)
NSTEMI	4 (14%)
STEMI	4 (14%)
Yes	1 (3·6%)
Familial History	10 (36%)
Creatinine (*μ*mol/L)	71 (2–118)
Cholesterol (mmol/L)	4 (3–127)
LDL-C (mmol/L)	90 (80–121)
HDL-C (mmol/L)	1 (1–24)
Triglycerides (mmol/L)	3 (1–160)
C-reactive protein (mg/dL)	
Unknown	7 (25%)
Negative	12 (43%)
Positive	9 (32%)
Hemoglobin (g/dL)	14·75 (12·57–16·33)

### Identified variants in the cohort

**Figure 1:**
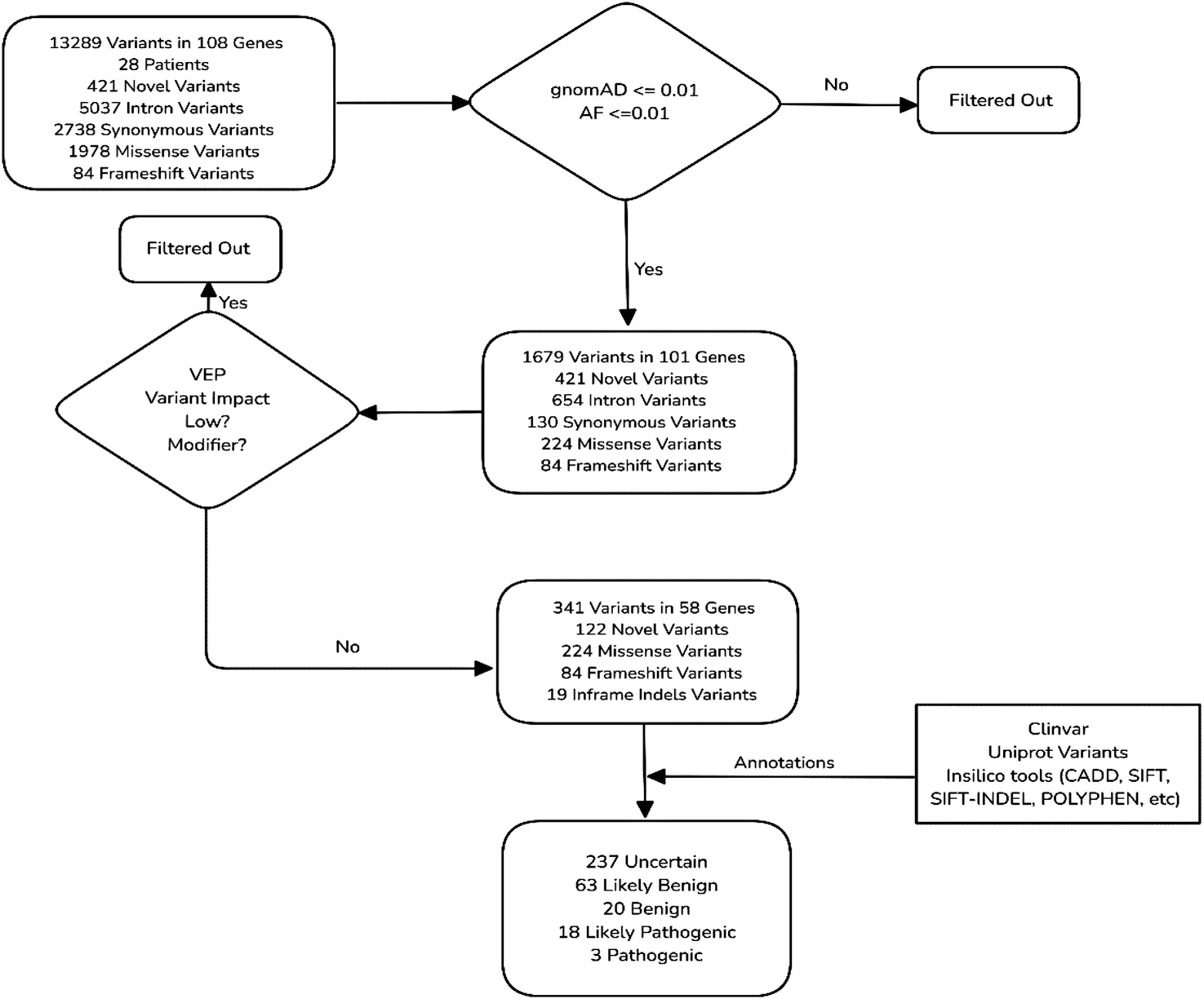
Workflow illustrating the steps involved in identifying, filtering, and classifying variants associated with sudden unexplained death or cardiac events through whole-exome sequencing.

**Figure 2:**
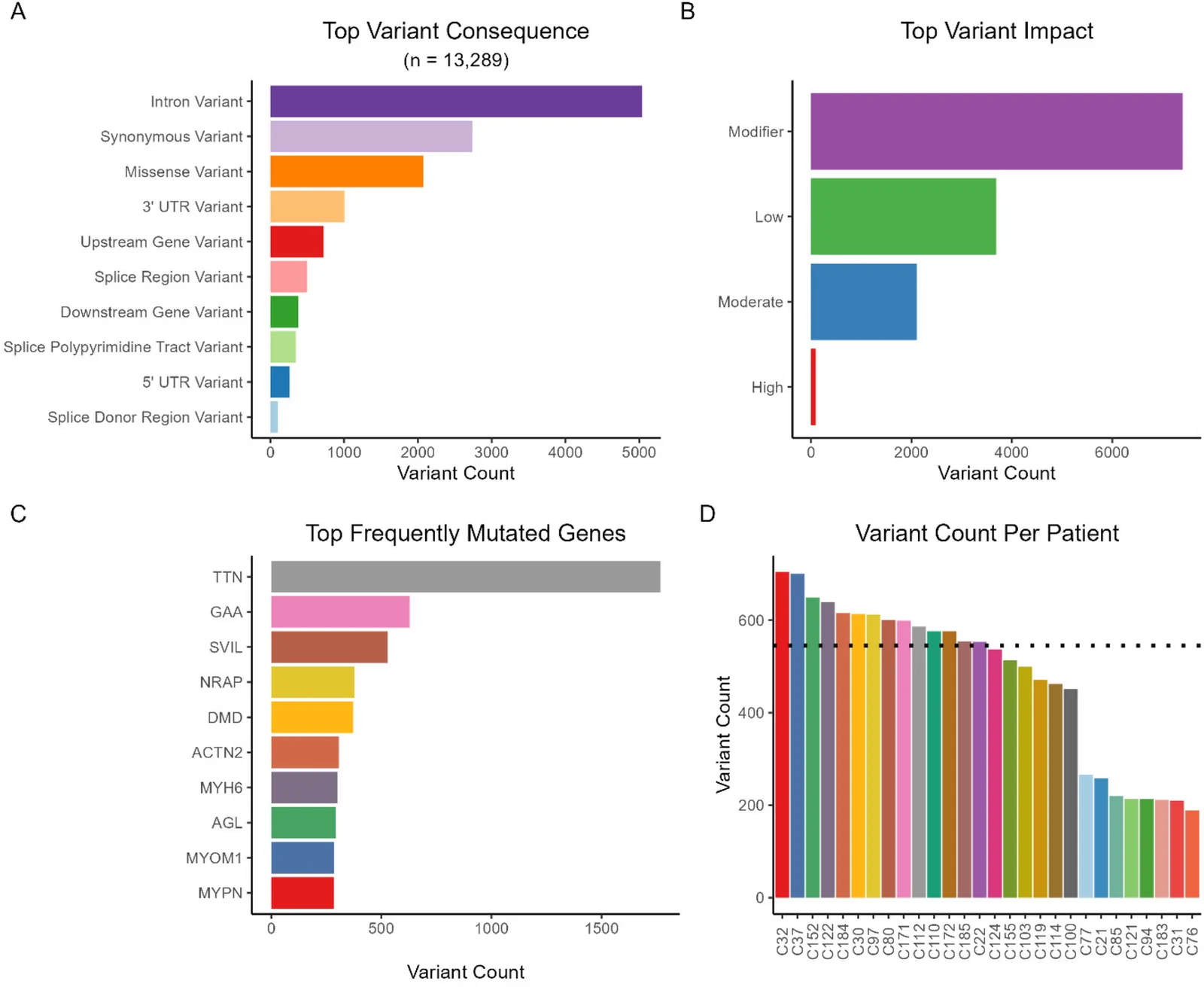
Overview of variant analysis results. **(A)** Distribution of variant types (e.g., missense, indel, etc.) **(B)** Categorization of variants based on VEP impact **(C)** Frequency of top mutated genes **(D)** Variant count distribution across samples.

A total of 108 genes, overlapping with the GenePanel, were identified with variants in our whole-exome sequencing analysis. This analysis yielded 13,289 variants across the cohort, with a median of 545 variants per patient. The majority of these variants were either intronic (38%) or synonymous (21%). Additionally, 1,978 missense variants, 1,003 3′ UTR variants, 262 5′ UTR variants, and 84 frameshift variants were identified. Notably, 421 variants (3%) were novel and not previously reported in public databases such as dbSNP or COSMIC. Based on VEP impact categorization, 92 variants were classified as high impact, 3,688 as low impact, 2,106 as moderate impact, and 7,403 as modifier impact. The most frequently mutated genes were TTN, followed by GAA, SVIL, NRAP, and DMD. Figure 1 illustrates the methodology used in the study to identify the candidate variants. Figure 2 provides the key summary of the identified variants in the study cohort.

### Candidate variant detection and classification

**Figure 3:**
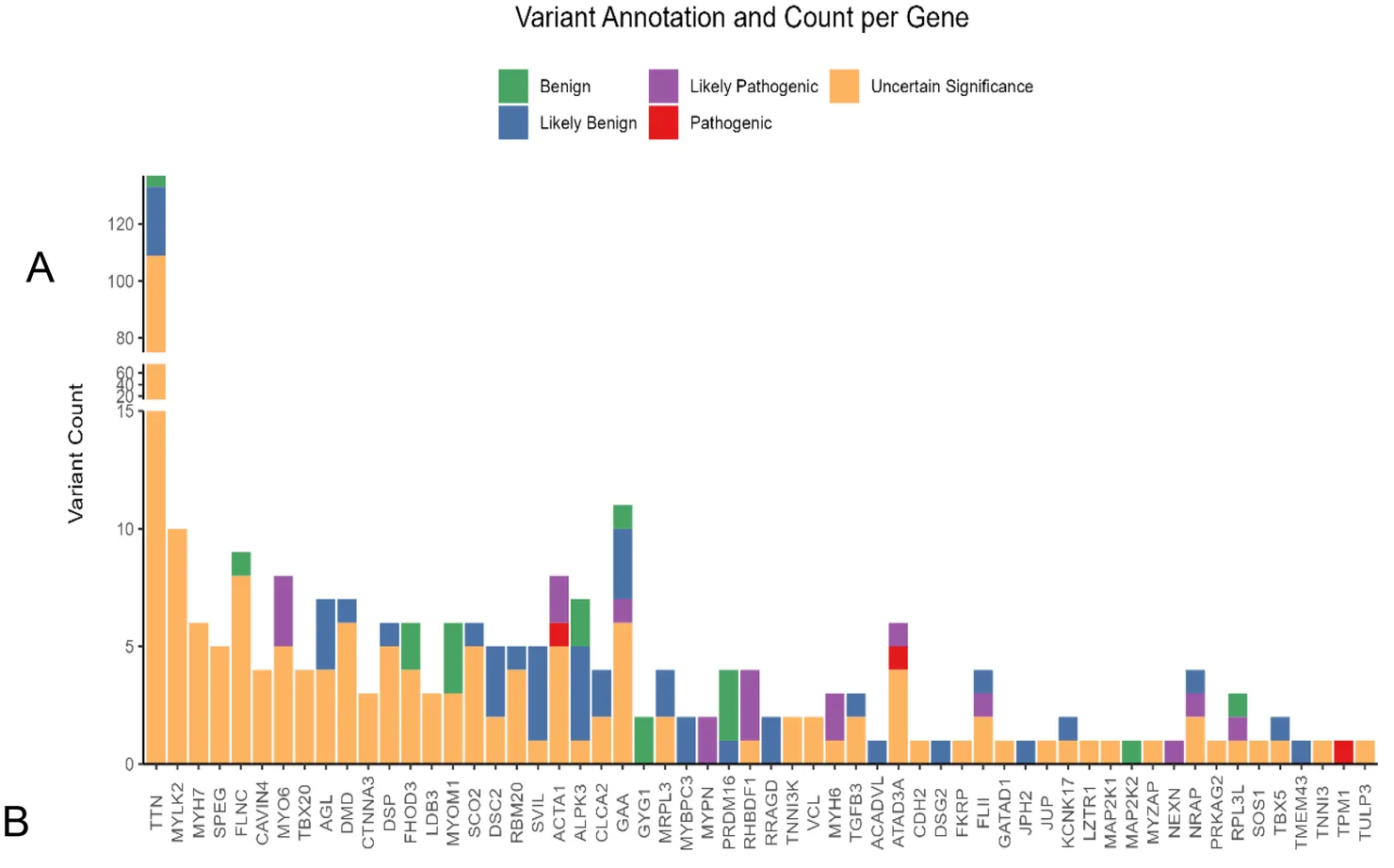
Candidate variant classification and co-occurrence **(A)** distribution of variant classifications (e.g., benign, likely benign, pathogenic, etc.) across top genes **(B)** Co-occurrence of top mutated genes in individual sample.

After filtering variants based on population frequency and VEP impact, 341 variants in 58 genes were identified. Of these, 122 variants (36%) were novel, not previously reported in public databases. The majority of the variants were missense (66%), followed by frameshifts (25%) and in-frame indels (5%). The remaining 4% were nonsense, splice site, and other protein-altering variants. To assess the clinical significance of these candidate variants, we annotated them using ClinVar, UniprotVar, and various in-silico prediction tools. The classification of these variants revealed a predominance of variants of unknown significance (VUS, 237), followed by likely benign (LB, 63), benign (B, 20), likely pathogenic (LP, 18), and pathogenic (P, 3) variants. TTN emerged as the most frequently mutated gene, being present in 96% of patient samples. Other frequently mutated genes included FLNC (29%), AGL (25%), ALPK3 (21%), and DSP (21%). Figure 3 provides a detailed overview of the candidate variants detected in each individual sample.

### Rare and potentially pathogenic variant identification

This study identified 21 candidate variants classified as either pathogenic (*n* = 3) or likely pathogenic (*n* = 18) within a cohort of 12 patients with cardiovascular disease. Each patient harbored at least one such variant. A majority of the identified variants (*n* = 18) were unique to individual patients. Two variants, however, were recurrent: MYO6 (rs551348450; ENST00000369977.8:c.2751dup) was observed in three patients, and MYPN (rs71534278; ENST00000358913.10:c.3335C>T) was found in two patients. All identified variants were heterozygous.

Among the identified likely pathogenic variants, eight novel alterations were detected in the GAA, MYH6, NEXN, ATAD3A, RHBDF1, and ACTA1 genes. Notably, these specific genomic coordinates are absent from major population and clinical databases, such as dbSNP, ClinVar, gnomAD, and the 1,000 Genomes Project. Comprehensive literature searches further confirmed that these variants have not been previously reported in any peer-reviewed case reports or cohort studies.

All 21 variants met the American College of Medical Genetics and Genomics (ACMG) criteria for PM2 classification, indicating a very low (<1%) or absent frequency in population databases. Further ACMG classification revealed additional supporting evidence for pathogenicity for specific variants. The MYO6 (ENST00000369977.8:c.2751dup), NEXN (ENST00000334785.12:c.175G>T), and ATAD3A (ENST00000378756.8:c.274A>T) variants were assigned PVS1 classification due to their nature as null variants (nonsense, frameshift, canonical splice sites, initiation codon, single or multi-exon deletion) in genes where loss-of-function (LOF) is a known mechanism of disease.

Missense variants identified in TPM1 (ENST00000403994.9:c.574G>A), GAA (ENST00000302262.8:c.1559A>T), and ACTA1 (ENST00000366684.7:c.984G>C; ENST00000366684.7:c.989A>T; ENST00000366684.7:c.983A>T) were classified as PM1 due to their presence in known hotspot regions. Variants in NRAP (ENST00000359988.4:c.919G>A), RHBDF1 (ENST00000262316.10:c.1880C>T), MYH6 (ENST00000405093.9:c.3710T>G;ENST00000405093.9:c.4865G>A),FLII (ENST00000327031.9:c.1841A>G), and RPL3L (ENST00000268661.8:c.34G>A) received PM2 and PP3 classifications, as multiple lines of computational evidence suggested a deleterious effect on the respective gene or gene product. Although TTN was the most frequently mutated gene across the cohort, no TTN variants met the ACMG and in silico consensus criteria required for a pathogenic or likely pathogenic designation. All final prioritized pathogenic variants were restricted to non-TTN loci.

A detailed summary of the identified unique pathogenic and likely pathogenic variants within this cohort is provided in Table 2. The detailed clinical phenotype of each individual patient cross-referenced with their respective pathogenic or likely pathogenic variants is provided in Supplementary Table S1.

**Table 2:** Key pathogenic and likely pathogenic variants identified in association with sudden cardiac events.

Gene	Variant ID	HGVSc	HGVSp	Variant Type	ACMG^a^ Criteria	Zygosity^b^	Annotation
*MYO6*	rs551348450	ENST00000369977.8:c.2751dup	ENSP00000358994.3:p.Gln918ThrfsTer24	Frameshift Variant	PVS1, PM2	Het	Likely Pathogenic
*NRAP*	rs911016723	ENST00000359988.4:c.919G>A	ENSP00000353078.3:p.Gly307Arg	Missense Variant	PM2, PP3	Het	Likely Pathogenic
*MYPN*	rs71534278	ENST00000358913.10:c.3335C>T	ENSP00000351790.5:p.Pro1112Leu	Missense Variant	PM5, PM2, PP3	Het	Likely Pathogenic
*TPM1*	rs199476315	ENST00000403994.9:c.574G>A	ENSP00000385107.4:p.Glu192Lys	Missense Variant	PM1, PM2, PP3, PP5	Het	Pathogenic
*GAA*	Novel	ENST00000302262.8:c.1559A>T	ENSP00000305692.3:p.Asn520Ile	Missense Variant	PM1, PM2, PP3	Het	Likely Pathogenic
*RHBDF1*	rs375953787	ENST00000262316.10:c.1880C>T	ENSP00000262316.5:p.Thr627Met	Missense Variant	PM2, PP3	Het	Likely Pathogenic
*MYH6*	Novel	ENST00000405093.9:c.3710T>G	ENSP00000386041.3:p.Met1237Arg	Missense Variant	PM2, PP3	Het	Likely Pathogenic
*NEXN*	Novel	ENST00000334785.12:c.175G>T	ENSP00000333938.7:p.Glu59Ter	Stop Gained	PVS1, PM2	Het	Likely Pathogenic
*ATAD3A*	Novel	ENST00000378756.8:c.274A>T	ENSP00000368031.3:p.Lys92Ter	Stop Gained	PVS1, PM2	Het	Likely Pathogenic
*RHBDF1*	Novel	ENST00000262316.10:c.147T>G	ENSP00000262316.5:p.Ser49Arg	Missense Variant	PM2, PP3	Het	Likely Pathogenic
*RHBDF1*	rs762900465	ENST00000262316.10:c.146G>A	ENSP00000262316.5:p.Ser49Asn	Missense Variant	PM2, PP1	Het	Likely Pathogenic
*FLII*	rs934095393	ENST00000327031.9:c.1841A>G	ENSP00000324573.4:p.Asp614Gly	Missense Variant	PM2, PP3	Het	Likely Pathogenic
*RPL3L*	rs146294352	ENST00000268661.8:c.34G>A	ENSP00000268661.7:p.Gly12Arg	Missense Variant	PM2, PP3	Het	Likely Pathogenic
*ATAD3A*	rs1057517686	ENST00000378756.8:c.1582C>T	ENSP00000368031.3:p.Arg528Trp	Missense Variant	PM2, PP3, PP5	Het	Pathogenic
*ACTA1*	rs398122936	ENST00000366684.7:c.984G>C	ENSP00000355645.3:p.Lys328Asn	Missense Variant	PM1, PM2, PP3, PP5	Het	Pathogenic
*ACTA1*	Novel	ENST00000366684.7:c.989A>T	ENSP00000355645.3:p.Lys330Met	Missense Variant	PM1, PM2, PP2, PP3	Het	Likely Pathogenic
*ACTA1*	Novel	ENST00000366684.7:c.983A>T	ENSP00000355645.3:p.Lys328Met	Missense Variant	PM1, PM2, PP2, PP3	Het	Likely Pathogenic
*MYH6*	Novel	ENST00000405093.9:c.4865G>A	ENSP00000386041.3:p.Gly1622Glu	Missense Variant	PM2, PP3	Het	Likely Pathogenic

a PM1 assigned due to localization within the actin domain of *ACTA1*, lysosomal alpha-glucosidase domain of *GAA,* and tropomyosin alpha-1 chain domain of TPM1.

b Heterozygosity.

### Functional characterization of pathogenic variants

**Figure 4:**
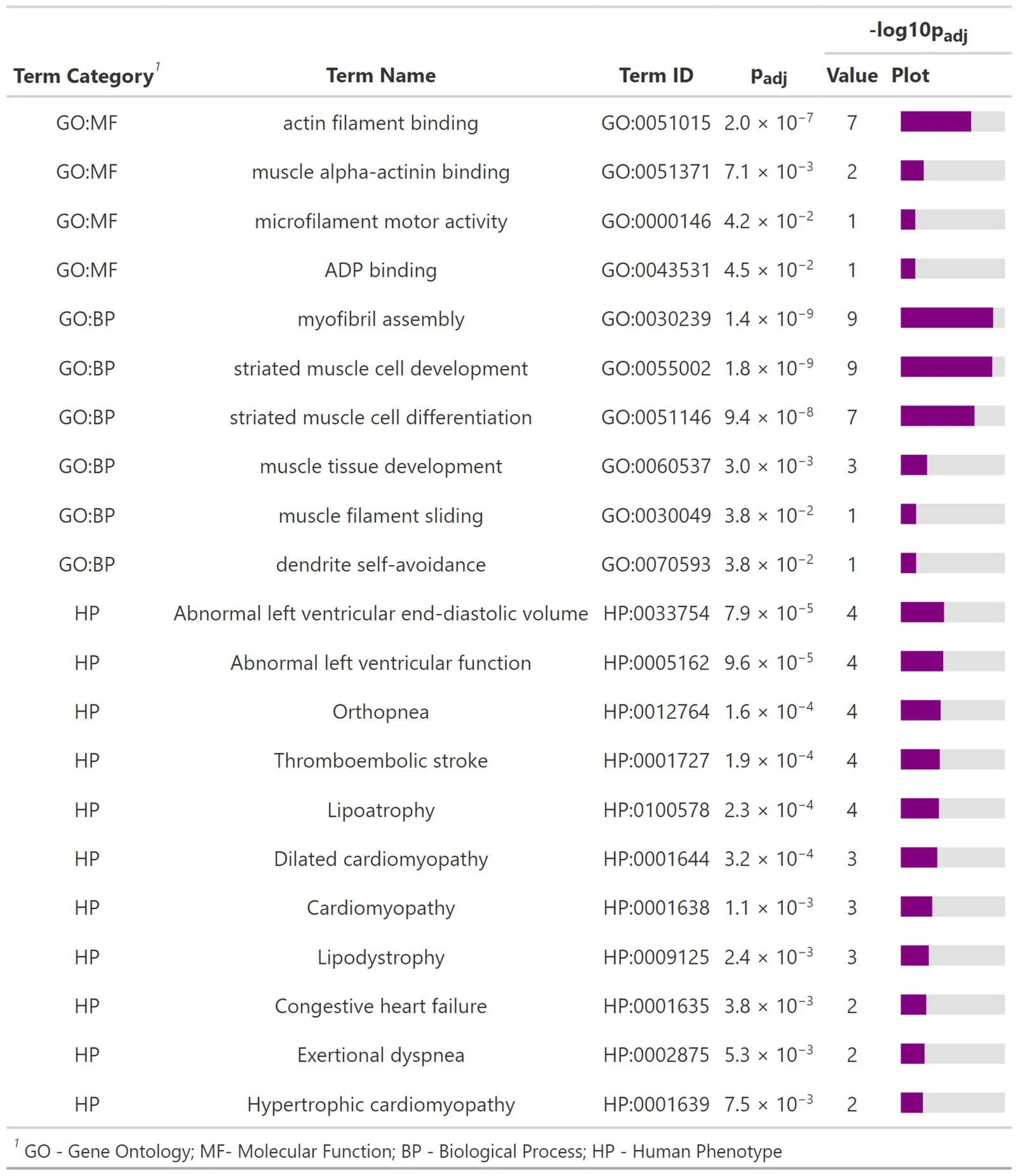
Gene ontology (GO) and human phenotype ontology (HPO) enrichment analysis results, highlighting significant terms and their corresponding adjusted *p*-values.

To delve deeper into the functional implications of the identified pathogenic and likely pathogenic variants, we conducted a comprehensive Gene Ontology (GO) and Human Phenotype Ontology (HPO) enrichment analysis of the genes harboring these variants. The GO analysis revealed significant enrichment in specific molecular functions and biological processes. In terms of molecular function (GO:MF), the genes were enriched for actin filament binding (p_adj_ = 2.0 × 10^−7^) and muscle alpha-actinin binding (p_adj_ = 7.1 × 10^−3^). Regarding biological processes (GO:BP), the genes were enriched for myofibril assembly (p_adj_ = 1.4 × 10^−9^) and striated muscle cell development (p_adj_ = 1.8 × 10^−9^). These findings indicate that the identified variants may disrupt fundamental processes involved in cardiac muscle development and function. The HPO enrichment analysis provided insights into the potential clinical manifestations associated with these variants. Notably, the genes were enriched for several cardiovascular phenotypes, including abnormal left ventricular end-diastolic volume (p_adj_ = 7.9 × 10^−5^), orthopnea (p_adj_ = 1.6 × 10^−4^), thromboembolic stroke (p_adj_ = 1.9 × 10^−4^), lipoatrophy (p_adj_ = 2.3 × 10^−4^), and dilated cardiomyopathy (p_adj_ = 3.2 × 10^−4^). These findings suggest that the identified variants may contribute to the development of diverse cardiovascular disorders. Figure 4 provides a visual representation of the enriched GO and HPO terms, highlighting the functional impact of the identified pathogenic variants.

## Discussion

Genome-wide association studies (GWAS) have been instrumental in identifying common genetic variants associated with CAD risk, with heritability estimates of 40%–50% attributed to common alleles at many loci (7). However, GWAS approaches have inherent limitations in detecting rare, high-impact variants that may significantly influence disease severity, myocardial resilience, or clinical outcomes in individual patients (34, 35). These rare variants, typically with allele frequencies <1%, are often missed by GWAS due to insufficient statistical power and the limitations of imputation-based genotyping arrays. Whole-exome sequencing overcomes these constraints by providing comprehensive, unbiased interrogation of protein-coding regions, enabling the discovery of rare and novel variants with potentially large effect sizes (36). While traditional genetic investigations of CAD have focused on canonical pathways in lipid metabolism, plaque stability, and systemic inflammation, WES allows exploration of variants in genes that may modulate myocardial structural integrity and electrical stability, factors that could lower the threshold for severe ischemic complications in patients with obstructive CAD. In an attempt to capture these potential modifiers, we have employed the Genomics England PanelApp's “Sudden Unexplained Death/Cardiac Event” super-panel, an integrated framework encompassing eight clinically curated cardiovascular sub-panels, including hypertrophic cardiomyopathy, dilated and arrhythmogenic cardiomyopathy, and various cardiac channelopathies. As mentioned such an approach allowed us to evaluate the presence of subclinical sarcomeric, desmosomal, and ion-channel variants that may influence phenotypic severity in our CAD cohort, extending beyond conventional atherosclerotic risk assessment.

An interesting finding of our study is the identification of variants in genes traditionally associated with primary cardiomyopathies such as *TTN*, *MYH6*, *NEXN*, *ACTA1*, *FLNC*, *ALPK3* and *DSP* within a cohort of patients with obstructive coronary artery disease. This observation raises an important conceptual question about the genetic overlap between coronary atherosclerosis and myocardial structural disorders. Particularly since the CAD and cardiomyopathies have been usually studied as distinct clinical entities, with CAD attributed primarily to atherosclerotic vascular disease and cardiomyopathies to intrinsic myocardial dysfunction (37). However, our findings suggest that these conditions may share genetic determinants that influence myocardial resilience within the ischemic stress. It is hypothesised that in patients with significant coronary stenosis, subtle genetic alterations such as those in sarcomeric proteins, desmosomal components, or cytoskeletal elements could predispose to adverse myocardial remodeling, arrhythmogenesis, or reduced tolerance to ischemia; mechanisms that may explain why some patients with similar coronary anatomy experience more severe clinical outcomes than others.

The high frequency of *TTN* variants (96% of patients) in our cohort needs a specific clarification. *TTN* encodes titin, the largest protein in the human body and a critical component of the sarcomere, functioning as a biological spring and structural scaffold (38). While pathogenic *TTN* variants are well-established causes of dilated cardiomyopathy (39), the prevalence in our CAD cohort likely reflects the gene's enormous size and its inherent propensity for accumulating rare background variation. Our stringent filtering pipeline successfully distinguished incidental *TTN* variants from clinically relevant alterations as no *TTN* variants met our combined ACMG and *in silico* consensus criteria for pathogenic classification. This approach is critical to avoid over-interpretation of *TTN* variants in non-cardiomyopathy populations, as up to 1%–3% of healthy individuals may harbor rare *TTN* variants. Beyond *TTN*, we observed substantial mutation frequencies in *FLNC* (29%), *AGL* (25%), *ALPK3* (21%), and *DSP* (21%). Filamin C (FLNC) maintains sarcomere structural integrity by crosslinking actin filaments (40), and FLNC variants are enriched in dilated cardiomyopathy (1%–4.5%) and hypertrophic cardiomyopathy (1.3%–8.7%) cohorts (41). ALPK3, encoding *α*-kinase 3, is a muscle-specific pseudokinase whose loss-of-function variants are associated with unique cardiomyopathy phenotypes in both children and adults (42, 43). Desmoplakin (DSP), a crucial desmosomal protein for cardiomyocyte cell-to-cell adhesion (44), has been linked to arrhythmogenic cardiomyopathy and left ventricular dysfunction, with DSP variant carriers facing significantly elevated rates of sustained ventricular arrhythmias and heart failure hospitalizations (45).

After applying strict population frequency filters (≤1% in gnomAD and 1,000 Genomes), VEP impact criteria (HIGH or MODERATE), and comprehensive in silico prediction consensus, we identified 21 variants classified as either pathogenic (*n* = 3) or likely pathogenic (*n* = 18) across 12 patients in our cohort. Each patient harbored at least one such variant, with all variants occurring in a heterozygous state. Most identified variants (*n* = 18) were unique to individual patients, suggesting a highly heterogeneous genetic landscape within our cohort. However, two recurrent variants were observed: *MYO6* (rs551348450; c.2751dup) in three patients, and *MYPN* (rs71534278; c.3335C>T) in two patients. This recurrence pattern, though limited by sample size, hints at potential founder effects or mutational hotspots that may warrant investigation in larger populations. All 21 variants met ACMG PM2 criteria, indicating very low or absent population frequency (<1%). Additional ACMG classifications provided further evidence of pathogenicity. Variants in *MYO6* (c.2751dup), *NEXN* (c.175G>T), and *ATAD3A* (c.274A>T) received PVS1 classification due to their nature as null variants (frameshift or stop-gained) in genes where loss-of-function is a known disease mechanism. Missense variants in *TPM1* (c.574G>A), *GAA* (c.1559A>T), and *ACTA1* (c.984G>C, c.989A>T, c.983A>T) were assigned PM1 classification due to localization in known functional hotspot regions. Additional PM2 and PP3 classifications supported the likely pathogenic designation for variants in *NRAP*, *RHBDF1*, *MYH6*, *FLII*, and *RPL3L*, based on multiple lines of computational evidence.

The eight novel likely pathogenic variants identified in this study span six genes with diverse but interconnected roles in cardiac structure and function. Their absence from all major genomic databases (dbSNP, ClinVar, gnomAD, 1,000 Genomes, COSMIC) and peer-reviewed literature demonstrates the value of WES in uncovering previously unrecognized genetic contributors to cardiovascular disease. These are specifically discussed as follows:

(i) *GAA (c.1559A*>*T; p.Asn520Ile)*

GAA encodes acid α-glucosidase (GAA), a lysosomal enzyme essential for glycogen degradation. While biallelic loss-of-function GAA mutations cause Pompe disease which is characterized by severe cardiomyopathy in the infantile form (46), the role of heterozygous GAA variants in cardiovascular disease has been less explored. Our identified variant localizes to the lysosomal alpha-glucosidase domain (PM1) and is predicted deleterious by multiple in silico tools. Previous studies have reported altered GAA expression in CAD patients (47), suggesting that even subtle reductions in GAA function could impair autophagy and contribute to myocardial vulnerability during ischemic stress. The identification of this novel heterozygous missense variant in a CAD patient raises the hypothesis that partial GAA deficiency may act as a modifier of cardiac function in the context of coronary artery disease.

(ii) *MYH6* (c.3710T>G; p.Met1237Arg and c.4865G>A; p.Gly1622Glu)

MYH6 encodes the alpha isoform of cardiac myosin heavy chain (*α*-MHC), a major component of the cardiac sarcomere. Genetic variations in MYH6 have been implicated in a spectrum of cardiovascular pathologies, including hypertrophic cardiomyopathy, dilated cardiomyopathy, and hypoplastic left heart syndrome (48). The two novel missense variants identified in our cohort, both predicted deleterious by multiple in silico tools, affect distinct domains of the α-MHC protein. Their presence in CAD patients without known cardiomyopathy suggests that partial functional impairment of *α*-MHC may reduce myocardial contractile reserve, potentially lowering the ischemic threshold during coronary insufficiency.

(iii) *NEXN (c.175G*>*T; p.Glu59Ter)*

NEXN encodes nexilin, an F-actin-binding protein localized at the cell-matrix interface of cardiomyocytes. NEXN has been identified as a susceptibility gene for CAD in Han Chinese populations (49), and variants in NEXN have been associated with right coronary artery fistulas and giant coronary aneurysms (50). The stop-gained variant identified here (PVS1 classification) results in premature protein truncation, likely leading to loss-of-function. Given nexilin's role in maintaining actin cytoskeleton integrity, this variant could impair the structural stability of cardiomyocytes, potentially increasing vulnerability to ischemic injury.

(iv) *ATAD3A* (c.274A>T; p.Lys92Ter)

ATAD3A encodes a mitochondrial membrane protein involved in mitochondrial dynamics and cholesterol metabolism. Genetic variations in ATAD3A have been linked to mitochondrial cardiac failure and hypertrophic cardiomyopathy (51). The stop-gained variant identified in our cohort (PVS1 classification) likely results in loss-of-function, potentially compromising mitochondrial function in cardiac myocytes. In the context of CAD, impaired mitochondrial energetics could reduce myocardial tolerance to ischemia, exacerbating injury during coronary insufficiency.

(v) *RHBDF1* (c.1880C>T; p.Thr627Met and c.147T>G; p.Ser49Arg)

RHBDF1 encodes a rhomboid protein, a conserved superfamily of polytopic membrane proteins involved in various cellular processes. Homozygous RHBDF1 variants have been identified in studies of consanguineous cardiomyopathy families (51). The two novel heterozygous missense variants identified in our cohort, both predicted deleterious, suggest that even monoallelic RHBDF1 alterations may contribute to cardiovascular susceptibility. The functional significance of RHBDF1 in the heart remains incompletely understood, but its emerging association with cardiomyopathy warrants further studies.

(vi) *ACTA1* (c.984G>C; p.Lys328Asn, c.989A>T; p.Lys330Met, c.983A>T; p.Lys328Met)

ACTA1 encodes the *α*-skeletal isoform of actin, a protein traditionally associated with skeletal muscle function. However, recent evidence has linked ACTA1 variants to dilated cardiomyopathy (52), suggesting a cardiac role for this isoform. Our three novel variants, all localized to known hotspot regions (PM1), are predicted pathogenic by multiple in silico tools. The identification of three distinct ACTA1 variants in our cohort, each in a different patient is notable and suggests that ACTA1 may represent a previously underappreciated CAD modifier gene. Actin is a fundamental component of the cardiac sarcomere, and disruptions in actin function could directly impair contractile function and myocardial resilience.

Notably, the genes harboring novel variants converge on common biological pathways that relate to caridiac function: sarcomeric structure and function (*MYH6*, *NEXN*, *ACTA1*), mitochondrial energetics (*ATAD3A*), lysosomal function (*GAA*), and membrane-associated signalling (*RHBDF1*). This convergence suggests that multiple pathways may contribute to myocardial vulnerability in CAD patients, and that individual patients may harbor distinct genetic susceptibilities. Moreover, the consistency across GO and HPO analyses from molecular function to biological process to clinical phenotype provides some convincing evidence that the identified variants are not merely statistical noise but likely contribute to genuine biological dysfunction with potential clinical consequences that may lower the threshold for ischemic injury in CAD.

## Limitations

Our study has several limitations, with the most significant being the small sample size of 28 patients. Although this cohort allowed for a detailed exploratory evaluation of rare exomic variants through rigorous bioinformatics filtering, the limited sample size reduces the generalizability of our findings and diminishes the statistical power to identify broader monogenic associations. Future prospective, large-scale, multi-center studies are necessary to validate these novel variants in more diverse CAD populations. Furthermore, while our pipeline utilized strict global allele frequency cutoffs (≤1%) via gnomAD and the 1,000 Genomes Project, the current cohort was not filtered against localized regional databases such as the Greater Middle East (GME) Variome or Qatar Genome Programme. Consequently, we cannot entirely rule out the presence of population-specific rare polymorphisms. Accessing and cross-referencing local reference databases represents a critical target for our ongoing validation studies. Additionally, orthogonal wet-lab confirmation (e.g., Sanger sequencing) and familial segregation analyses were not performed, which represents a limitation of this exploratory analysis. Future work will focus on performing targeted Sanger verification and familial screening for the 8 identified novel likely pathogenic variants to definitively trace their inheritance patterns.

## Conclusion

The study has utilized whole exome sequencing to identify both known and novel gene variants that may contribute to disease risk, facilitating a deeper understanding of the genetic landscape of CAD. This high-resolution approach allows the detection of pathogenic and likely pathogenic variants in critical genes associated with cardiovascular health and provides evidence of functional significance, thereby elucidating complex interactions between genetic factors and enhancing understanding of disease mechanisms.

## Data Availability

The original contributions presented in the study are included in the article/Supplementary Material, further inquiries can be directed to the corresponding author.
